# Hepatocellular carcinoma cell lines retain the genomic and transcriptomic landscapes of primary human cancers

**DOI:** 10.1038/srep27411

**Published:** 2016-06-07

**Authors:** Zhixin Qiu, Keke Zou, Liping Zhuang, Jianjie Qin, Hong Li, Chao Li, Zhengtao Zhang, Xiaotao Chen, Jin Cen, Zhiqiang Meng, Haibin Zhang, Yixue Li, Lijian Hui

**Affiliations:** 1 State Key Laboratory of Cell Biology, Shanghai Institute of Biochemistry and Cell Biology, Shanghai Institutes for Biological Sciences, Chinese Academy of Sciences, 200031 Shanghai, China; 2 Key Lab of Computational Biology, CAS-MPG Partner Institute for Computational Biology, Shanghai Institutes for Biological Sciences, Chinese Academy of Sciences, 200031 Shanghai, China; 3 Department of Minimally Invasive Therapy, Collaborative Innovation Center for Cancer Medicine, Fudan University Shanghai Cancer Center, Department of Oncology, Shanghai Medical College, Fudan University, Shanghai, China, 200032 Shanghai, China; 4 Liver Transplantation Center, Key Laboratory of Living Donor Liver Transplantation of Ministry of Public Health, The First Affiliated Hospital of Nanjing Medical University, 210029 Nanjing, China; 5 Fifth Department of Hepatic Surgery, Eastern Hepatobilliary Surgery Hospital, Second Military Medical University, 200438 Shanghai, China; 6 University of Chinese Academy of Sciences, 100049 Beijing, China

## Abstract

Hepatocellular carcinoma (HCC) cell lines are useful *in vitro* models for the study of primary HCCs. Because cell lines acquire additional mutations in culture, it is important to understand to what extent HCC cell lines retain the genetic landscapes of primary HCCs. Most HCC cell lines were established during the last century, precluding comparison between cell lines and primary cancers. In this study, 9 Chinese HCC cell lines with matched patient-derived cells at low passages (PDCs) were established in the defined culture condition. Whole genome analyses of 4 HCC cell lines showed that genomic mutation landscapes, including mutations, copy number alterations (CNAs) and HBV integrations, were highly stable during cell line establishment. Importantly, genetic alterations in cancer drivers and druggable genes were reserved in cell lines. HCC cell lines also retained gene expression patterns of primary HCCs during *in vitro* culture. Finally, sequential analysis of HCC cell lines and PDCs at different passages revealed their comparable and stable genomic and transcriptomic levels if maintained within proper passages. These results show that HCC cell lines largely retain the genomic and transcriptomic landscapes of primary HCCs, thus laying the rationale for testing HCC cell lines as preclinical models in precision medicine.

In past decades, cancer cell lines have played important roles in cancer studies for both dissecting molecular mechanisms and developing new drugs^1^. The large cell line-based platforms, such as NCI-60 and Cancer Cell Line Encyclopedia (CCLE), have been used to represent the genetic heterogeneity of cancer cells and to identify biomarkers allowing patient stratification in precision medicine^1^,^2^. The rationale for using cancer cell lines as an experimental model is that cancer cell lines retain the hallmarks of primary cancer cells^3^. However, there are concerns about whether cancer cell lines could faithfully represent the matched primary cancer cells in terms of genomic mutations and transcriptomic profiles. For example, cell lines may gain additional mutations during long-term culture, which renders them no longer representative of the primary cancers from which they were derived^4^. However, because most cancer cell lines were established a long time ago, it is difficult to characterize the degree to which these cell lines represent their matched primary cancers.

To that end, several studies have generated new cancer cell lines and compared these cell lines to their matched primary cancers. Data from these studies provide evidence supporting the similarity between cancer cell lines and primary cancers^3^. It has been reported that *TP53* mutations were retained in 53 out of 62 pairs of the matched leukemia cell lines and primary cancer cells^5^. Another study showed that glioblastoma cell lines retained the same *CDKN2A* homozygous deletions with the original tumors^6^. Morphological features, aneuploidy, and immunostaining of HER2 and p53 were found to be consistent between lung cancer cell lines and matched cancers^7^. Copy number profiles of primary cancers were largely preserved in cell lines when compared to their primary breast cancers^8^ and glioblastomas^9^. These results demonstrated that cancer cell lines and matched primary cancers are similar in some key phenotypic and molecular characteristics. However, these studies only examined a few mutations and copy number alterations. There have been very few analyses of transcriptomic similarities between cell lines and matched primary cancers. Besides established cell lines, it has been proposed that cancer cells at early passages during cell line establishment (around passage 5, also called patient-derived cells, PDC) may faithfully represent primary cancers^10^,^11^,^12^. Indeed, PDCs were found similar to those of the primary cancers in terms of key gene mutations, copy number profiles and drug responses^11^. Apparently, PDCs would be the valuable *in vitro* intermediate to assess genetic changes in cell lines. However, there has yet to be any careful characterization between cancer cells at early passages and established cell lines.

Hepatocellular carcinoma (HCC), the major type of liver cancer, has emerged as the second most common cause of cancer-related death^13^. Sorafenib is the only approved targeted drug for advanced HCC^14^. It is highly desirable to develop new drugs for this dreadful disease. Data from the large-scale sequencing studies have identified the genomic heterogeneity of HCC, including mutations in *TERT* promoter, *TP53* and *CTNNB1*^15^. Notably, many HCC patients harbor genetic alterations in genes potentially targetable with clinically approved drugs^16^. These genomic findings must be translated into novel therapeutic strategies through functional characterizations using experimental models that faithfully recapitulate cancerous features of primary HCCs. So far, cancer cell lines for HCC are very limited; there are only about 30 publically available HCC cell lines, and genetic characteristics of these cell lines is largely undetermined^2^. Importantly, their representativeness for primary HCCs is unknown, because all HCC cell lines were established several decades ago, precluding a direct comparison to their primary HCCs. For this reason, it is critical to establish new HCC cell lines and to validate whether these cell lines faithfully represent their matched HCCs at genomic and transcriptomic levels.

In this study, 9 cell lines from Chinese HCC patients were established. Whole genome sequencing (WGS) was performed in 4 matched cell lines, PDCs and HCCs and RNAseq was performed to characterized transcriptome in 9 pairs of cell lines and PDCs and 6 matched primary HCCs. Notably, comparison of cell lines and HCCs demonstrated that, during long-term *in vitro* culture, cell lines retain the genetic landscape of the matched primary HCCs. These data showed that HCC cell lines represent primary HCCs with high fidelity. Moreover, sequential analysis at different passages showed that HCC cell lines are similar to HCC cells of early passages at the genomic and transcriptomic levels, suggesting a comparable power of both cellular models to represent primary HCCs.

## Results

### Nine HCC cell lines were established from Chinese patients

**Figure 1:**
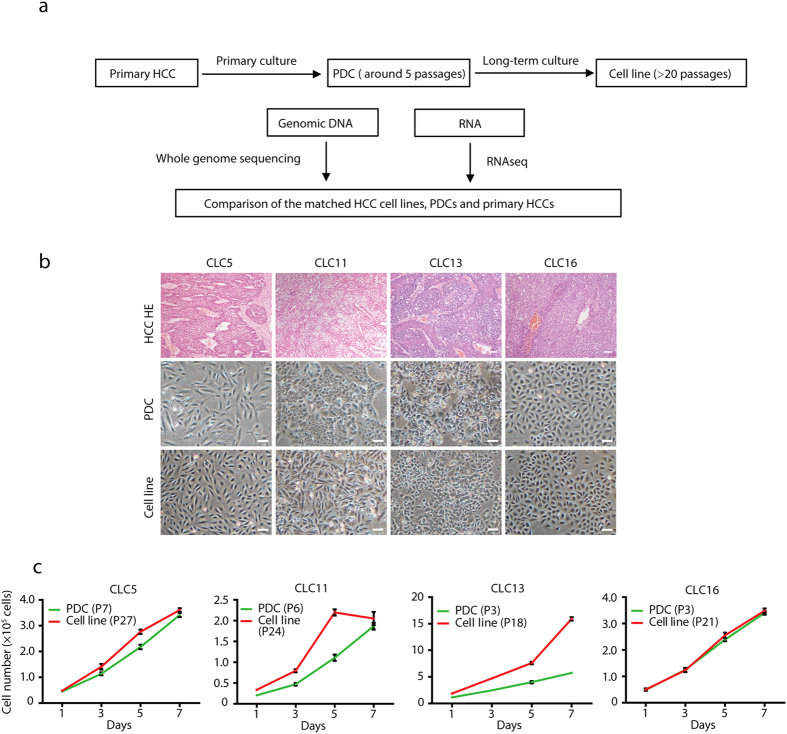
Establishment of nine hepatocellular carcinoma cell lines from Chinese patients. (**a**) Schematic outline for the primary culture of Chinese HCC specimens and establishment of HCC cell lines and PDCs. (**b**) Histology of primary HCCs characterized by Haematoxylin and eosin (HE) staining and morphologies of PDCs and cell lines. Scale bars: 100 μm. (**c**) Growth curves of the matched PDCs and cell lines. The passage numbers of cell lines or PDCs are indicated in the legends.

In order to perform a direct comparison of HCC cell lines and matched primary cancers, we established new cancer cell lines from HCC specimens of Chinese patients (Fig. 1a). A slightly modified method of cell line establishment was used based on published protocols^17^ (see methods for detail). Epithelial clones were picked out from the primary culture to enrich epithelial HCC cells. In total, 9 liver cancer cell lines were generated from 64 HCC samples with overall success rate of 14%, which was moderately higher than reported data^17^. The clinical information of HCC patients for 9 cell lines was summarized in Table S1. Eight out of nine patients were infected with hepatitis B virus (HBV) and one with hepatitis C virus (HCV).

All cell lines were cultured *in vitro* for more than 20 passages (passage 20–38). We obtained cells at passage 2–8 to represent early passages during the establishment of cell lines (Table S2). These cell lines were named Chinese Liver Cancer cell lines (CLCs). “T” and “PDC” were added after cell line names to indicate the matched primary HCCs and PDCs, respectively. All cell lines and matched HCCs were authenticated using short tandem repeat (STR) profiling to exclude cross contamination (Table S3). These cell lines grew as an adherent monolayer (Fig. 1b, Supplementary Fig. S1) with doubling time around 2–3 days (Fig. 1c). Hepatic origins were confirmed by expression of Albumin and AFP and, specifically, by HBV integration in cell lines from HBV-positive patients (Supplementary Fig. S1). These newly established cell lines made it possible to directly compare HCC cell lines to their matched primary HCCs.

### Global SNV patterns remain stable during cell line establishment

**Figure 2:**
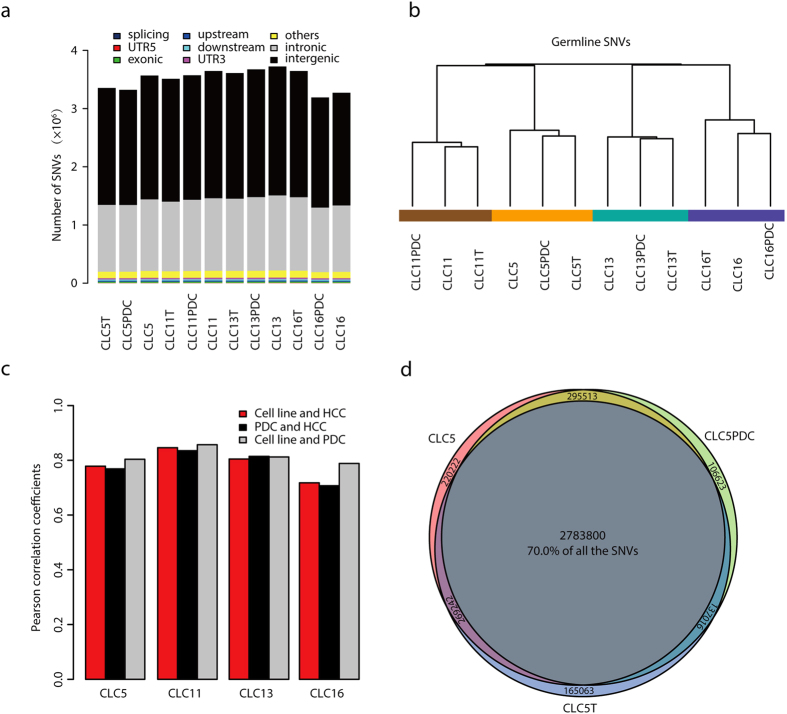
Whole genome SNV similarity among the matched cell lines, primary HCCs and PDCs. (**a**) Statistics of SNVs identified from whole genome sequencing data. UTR, untranslated region. (**b,c**) Unsupervised hierarchical clustering (**b**) and calculation of paired Pearson correlation coefficients (**c**) of 4 matched HCC cell lines, primary HCCs and PDCs using whole genome germline SNVs. (**d**) Venn diagram shows the overlapping of whole genome SNVs in the cell line, primary HCC and PDC of the patient CLC5.

To compare HCC cell lines with matched primary HCCs at the genome level, whole genome sequencing (WGS) was performed in 4 cell lines and their matched HCCs and PDCs to a mean mappable sequencing depth of 31.68 × (27.41–34.91×, Table S4). These four HCC patients were selected to represent the heterogeneity of primary HCCs in terms of age, tumor size, serum AFP level and immunohistochemical staining of Hep-1, a marker of differentiation (Table S1). On average, up to 93.88% of the genome was covered at ≥10× depth. About 3–4 million high-quality single nucleotide variations (SNVs) and insertion and deletion (InDel) variations were identified in each sample using Isaac Whole Genome Sequencing pipeline (Fig. 2a). More than 99% of the variations were in the noncoding regions, and 0.91–0.98% were in the exonic regions. These data allowed us to compare *in vitro* cells at different passages to their matched primary HCCs.

To determine whether global SNV patterns were stable during the establishment of cell lines, unsupervised hierarchical clustering was performed using whole genome germline SNVs (Fig. 2b). HCC cell lines and matched primary HCCs grouped together in the same clusters. Remarkably, Pearson correlation coefficients of the matched HCC cell lines, primary HCCs and PDCs were around 0.8 (Fig. 2c), suggesting a high correlation between HCC cell lines and primary HCCs. For example, in CLC5, 90% of SNVs were shared between the cell line and primary HCC (Fig. 2d). We would like to note that, due to the fact that not all of the genome was sequenced at a high depth by WGS, SNVs in the regions which were either not sequenced or sequenced at a low depth might be overlooked^18^. The actual number of shared SNVs among the matched cell lines, primary HCCs and PDCs may be higher than calculated here. Collectively, these results showed that global SNV patterns between the cell lines and matched HCCs were stable.

### HCC cell lines preserve landscapes of genomic alterations in primary HCCs

The progression of HCC is accompanied by extensive genomic alterations, such as somatic mutations, copy number alterations (CNAs), and HBV integrations^15^. HCC cell lines were examined to determine whether they retained the genomic alteration landscapes of primary HCCs. First, the protein-altering somatic mutations in the exome, including missense and nonsense mutations, were analyzed. Because mutations with low SNV calling quality scores might be overlooked^18^, protein-altering somatic mutations were also curated by recovering those identified in only some of the samples. For example, *TP53* mutations were identified in HCC cell lines, but did not pass the filter for SNV calling quality in matched primary HCCs in CLC 5, 11, 13 (Supplementary Fig. S2). To determine whether these *TP53* mutations exist in primary HCCs, mapping results were inspected manually and sequencing reads harboring TP53 mutations were counted to calculate variant allele frequencies (VAFs). *TP53* mutations were supported by sequencing reads in HCCs with VAFs greater than 90% (Supplementary Fig. S2). *TP53* mutations were further validated using Sanger sequencing in HCC cell lines, primary HCCs and PDCs (Supplementary Fig. S2). By manually checking the quality scores of SNV calling, a total of 294 protein-altering somatic mutation sites (7.9% of all sites) were curated in all the cell lines, primary HCCs and PDCs (Table S5).

**Figure 3:**
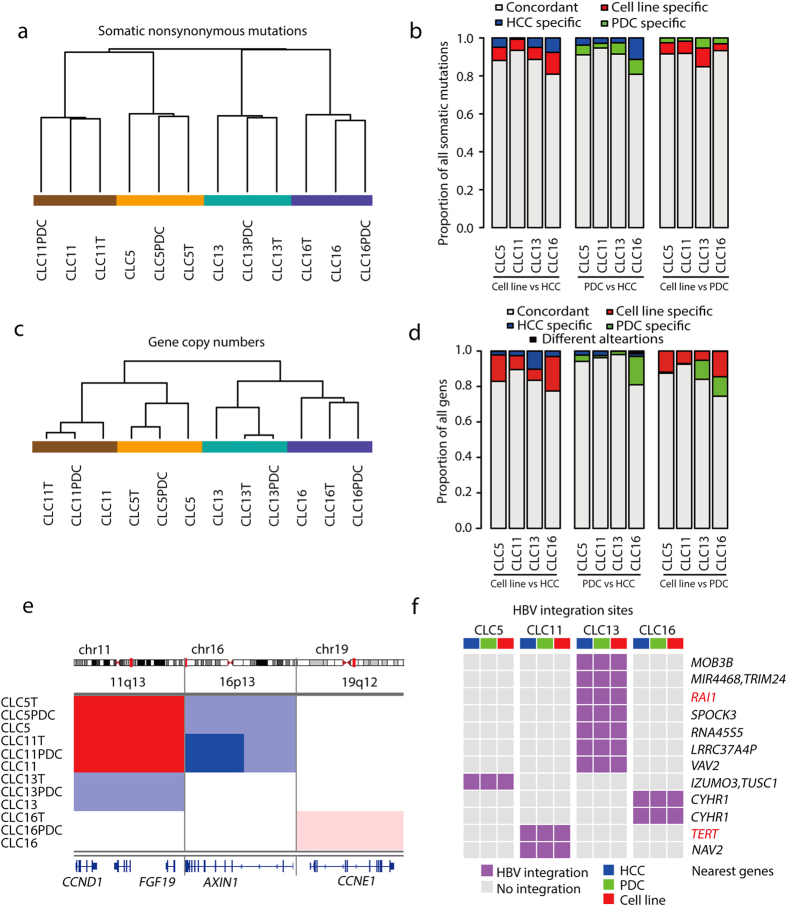
Comparison of genomic alteration landscapes. (**a,b**) Unsupervised hierarchical clustering (**a**) and paired comparison (**b**) of the matched cell lines, primary HCCs and PDCs using protein-altering somatic mutations. (**c,d**) Unsupervised hierarchical clustering (**c**) and paired comparison (**d**) of the matched cell lines, primary HCCs and PDCs using gene copy numbers. “Different alterations” means genes that are amplified and deleted in each sample in paired comparison, respectively. (**e**) Typical copy number alterations (CNAs). Red, amplification. Blue, deletion. Chr, chromosome. (**f**) Heatmap shows patterns of HBV integration sites. The genes nearest to the HBV integration sites are listed. Genes with HBV integration sites identified previously are in red.

A total of 232–377 putative protein-altering somatic mutations were eventually identified in each HCC cell line, primary HCC and PDC (Supplementary Fig. S3). Unsupervised hierarchical clustering using protein-altering somatic mutations showed the similarity of the matched cell lines, primary HCCs and PDCs (Fig. 3a). Paired analyses revealed that HCC cell lines shared 81–93% of protein-altering somatic mutations with matched primary HCCs (Fig. 3b, Supplementary Fig. S3). These results were consistent with findings of whole genome comparison of SNVs (Fig. 2), suggesting that HCC cell lines retained mutational spectra of the matched primary HCCs.

Previous studies showed that HCCs had extensive copy number alterations^16^,^19^. Next, we compared the CNA profiles calculated from sequencing depth using the Control-FREEC^20^. Unsupervised hierarchical clustering using gene copy numbers showed that the matched HCC cell lines, primary HCCs and PDCs were in the same clusters (Fig. 3c), indicating that their overall copy number profiles were comparable. On average, more than 80% of genes displayed consistent copy number status among pairs of matched HCC cell lines, primary HCCs and PDCs (Fig. 3d). We analyzed copy number changes at the chromosome arm level to reflect aneuploidy by GISTIC^21^. In line with analysis of gene copy numbers, 70~80% of chromosome arms showed consistent copy numbers between the matched cell lines and primary HCCs, indicating their overall similarities in aneuploidy (Supplementary Fig. S4). We examined typical CNAs reported in HCC patients^16^, and found these CNAs in primary HCCs were also largely presented in their matched cell lines (Fig. 3e). For example, CLC5 and CLC11 harbored amplification of 11q13 region containing *CCND1-FGF19* and homozygous deletion of 16p13 containing *AXIN1. CCNE1* was amplified in both CLC16 primary HCC and the matched cell line.

The 4 HCC cell lines analyzed by WGS were all derived from HBV-infected patients. HBV DNA promotes liver carcinogenesis by integration into the host genome and activation of cancer genes^13^,^15^. To determine HBV integration sites, whole genome sequencing reads were analyzed using Virus-Clip^22^. A total of 45 integration sites were detected (range from 1 to 10, Table S6). Of these sites, two had been reported previously, including the hotspot integration site in *TERT* gene promoter^23^,^24^. HBV integrations were retained in PDCs and cell lines (Fig. 3f), suggesting the similarity between matched cell lines, primary HCCs and PDCs. Collectively, these analyses of protein-altering somatic mutations, CNAs and HBV integrations demonstrated that HCC cell lines preserved genomic alteration landscapes of primary HCCs.

### Alterations in driver genes and druggable genes in primary HCCs are retained in cell lines

**Figure 4:**
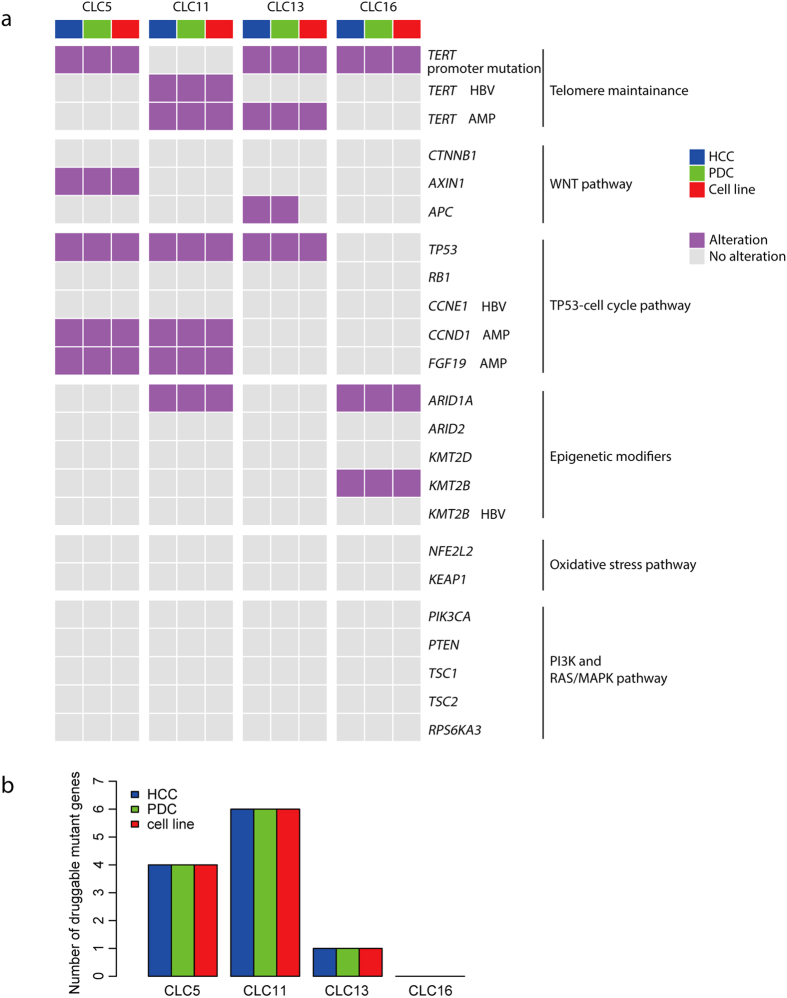
Comparison of driver gene alterations. (**a**) Heatmap shows landscapes of genetic alterations for HCC driver genes in the matched cell lines, primary HCCs and PDCs. Protein-altering somatic mutations, copy number alterations (CNAs) and HBV integrations were taken into consideration. The notes after gene symbol: AMP, amplification; DEL, deletion; HBV, HBV integration. No notes after gene symbol indicate protein-altering somatic mutations. (**b**) The number of druggable mutant genes in primary HCCs.

Recent studies of whole genome sequencing of HCCs have identified a panel of cancer driver genes, such as *TP53*, *CTNNB1* and *ARID1A*, which are critical in HCC development^15^. We next asked whether HCC cell lines also retained genomic alterations on driver genes in matched primary HCCs. In addition to *TP53*, mutations in *TERT* promoter, *AXIN1* and *APC* which differed across the matched cell lines, primary HCCs and PDCs, were also validated using Sanger sequencing (Table S5). Analysis of recently summarized driver gene alterations^15^ showed that these driver gene alterations were preserved among the HCC cell lines, primary HCCs and PDCs (Fig. 4a). Three HCC cell lines and matched primary HCCs harbored a common mutation site in *TERT* promoter located 124-bp upstream of the start codon, which accounts for 93% of *TERT* promoter mutations in HCC^23^. HCC cell line CLC11 and its matched HCC harbored an HBV integration into the *TERT* promoter. This pattern was in line with the mutually exclusive occurrence of the *TERT* promoter mutations and HBV integration in HCC^23^. *TP53* mutations (E68X, G105D and C238F), including 1 nonsense mutation and 2 missense mutations, were observed in 3 HCC cell lines and their matched primary HCCs (Fig. 4a). These *TP53* mutations were also presented in Catalogue of Somatic Mutations in Cancer (COSMIC), a database collecting somatic mutations in human cancers^25^. Mutations in *CTNNB1*, which is mutated in 11%–37% of primary HCCs^15^, were not detected in any of four HCC cell lines or matched HCCs. However, mutations in other components of Wnt pathway were identified. For example, nonsense mutations in *AXIN1* were detected in the cell line CLC5 and matched primary HCC. Interestingly, *APC* mutation was detected in primary HCC and PDC, but not in CLC13 cell line, which may be attributed to intratumoral heterogeneity and cell population selection during culture^5^. Among epigenetic modifiers, missense mutations in *ARID1A* and *KMT2B* were detected in two HCC cell lines and matched primary HCCs. Mutations in PI3K or MAPK pathways were not detected, likely reflecting their low mutation frequencies in HCC. We next estimated the VAFs of these driver mutations from whole genome sequencing and found that the VAFs remained comparable across the matched cell lines, primary HCCs and PDCs (Table S7).

We next analyzed whether alterations of druggable genes were retained in cell lines. By examining protein-altering mutations in druggable genes^26^, we found 11 potential druggable mutant genes in primary HCCs, which were also present in cell lines and PDCs (Fig. 4b, Table S8). Moreover, *CCND1*-*FGF19* amplification was present in 2 primary HCCs and cell lines (Fig. 3e). Amplification of *CCND1* is a possible biomarker to predict responses to CDK4/6 inhibitors (Palbociclib)^27^. FGF19 is also a potential target in HCC^28^. Although functional consequence of these alterations remained to be validated, it would be interesting to test inhibitors or antibodies targeting these genetic alterations in a large panel of HCC cell lines to evaluate their potential use in HCC. Druggable alterations with rare frequencies in HCC, such as EGFR, KRAS and PI3K, were identified neither in primary HCCs nor in cell lines. These results highlighted the translational implications of HCC cell lines by representing the matched primary HCCs in spectrums of genetic alterations in driver genes and druggable genes.

### Mutations emerge during cell line establishment

**Figure 5:**
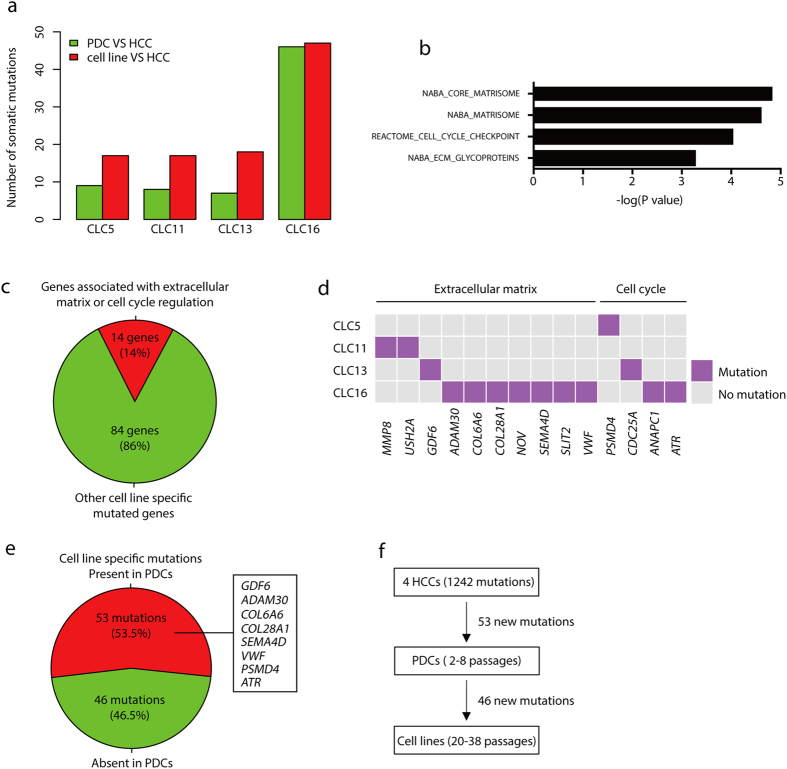
Characterization of cell line-specific protein-altering somatic mutations. (**a**) The number of cell line-specific and PDC-specific mutations compared to the matched primary HCCs. (**b**) Pathway enrichment analysis of genes affected by the cell line-specific mutations. (**c**) Pie chart shows the percentage of genes contributing to the pathway enrichment. (**d**) Heatmap shows mutant genes which contributed to the enrichment of extracellular matrix- or cell cycle-related pathways. (**e**) The percentage of cell line-specific mutations detected in the matched PDCs. Red indicates the presence in PDCs. Highlighted genes are involved in extracellular matrix and cell cycle. (**f**) Emergence of cell line-specific mutations. In these 4 cell lines, 53 mutations were already present in PDCs. Total mutations (46) were divided by total passages (91) in 4 patients to calculate the rate of mutation emergence from PDCs to cell lines.

Compared to the matched HCCs, there were 17–47 protein-altering mutations specific to each HCC cell line, representing 5.7–11.4% of total protein-altering somatic mutations (Fig. 5a). A total of 99 cell line-specific protein-altering mutations were identified in 98 genes (Table S9). All these cell line-specific mutations were unique to single HCC cell lines (Table S9), suggesting no common mutation in the establishment of 4 HCC cell lines. Among 99 mutations, only 5 were presented in COSMIC, including missense mutations in *PSMD4*, *TAPT1*, *AKR1D1*, *SNCAIP* and *SNX13*. However, frequencies of the 5 mutations were extremely low (no more than 2 cases) among more than 20,000 tested cancer samples in COSMIC database (Table S9), implicating that these mutations are unlikely key cancer driver mutations. Cell line-specific mutant genes were also compared to 572 known cancer genes in Cancer Gene Census database^29^ and driver genes identified by human HCC sequencing and TCGA pan-cancer project^15^,^30^. We found that 98 out of the 99 mutations did not occur in reported cancer driver genes, with only one exception of a nonsense mutation in *ATR* (L608X) in CLC16, which was not a candidate cancer driver gene in HCC^15^.

We next determined whether these cell line-specific mutations correlated with any pathways using Gene Set Enrichment Analysis^31^. We found that 84 out of 98 mutant genes (86%) were not significantly enriched in any pathways (Fig. 5c), suggesting that the majority of cell line-specific mutations occurred randomly. For the remaining 14 mutant genes (14%), enrichment in extracellular matrix-associated pathways and cell cycle-associated pathway was detected (Fig. 5b,c), although the enrichment confidence was low (Fig. 5b). Notably, each cell line acquired at least one mutant gene associated with extracellular matrix or cell cycle (Fig. 5d). These findings likely reflect the nature of *in vitro* culture, which contains specialized extracellular matrix and a high concentration of serum and growth factors in the medium^32^.

We also analyzed protein-altering mutations in PDCs, which provided an opportunity to characterize the generation of cell line-specific mutations during early stages of cell line establishment. We identified 7–46 additional protein-altering mutations in each PDC compared to matched primary HCC, which was about half of cell line-specific protein-altering mutations (Fig. 5a). About 53.5% (range from 18% to 85% in HCC cell lines) of cell line-specific mutations had already occurred in matched PDCs (Fig. 5e, Supplementary Fig. S5). No significant pathway enrichment was found using PDC-specific mutations. Intriguingly, eight mutations associated with extracellular matrix or cell cycle were already present in PDCs (Fig. 5e, Supplementary Fig. S5). These data showed that early-passage PDC cells gained around half of total new mutations *in vitro*, likely due to clonal selection of HCC cells during the culture. With passages increasing, additional mutations were gained at the rate of around 0.5 mutations per passage (Fig. 5f). Importantly, almost all these mutations occurred randomly and had little effect on driver genes and druggable genes.

### Gene expression patterns in primary HCCs are retained in cell lines

**Figure 6:**
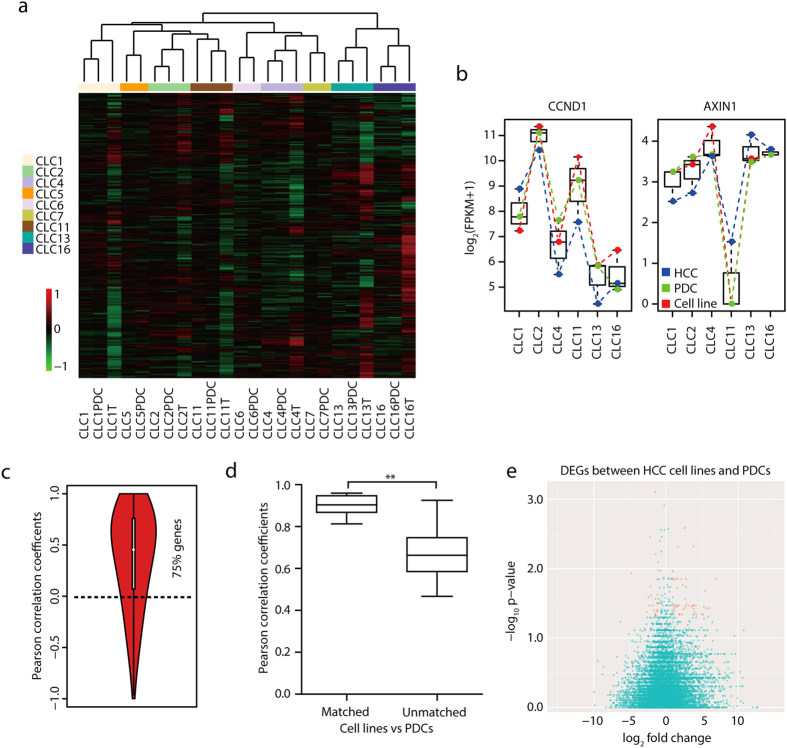
Transcriptome analysis during long-term *in vitro* culture. (**a**) Unsupervised hierarchical clustering of the matched cell lines, primary HCCs and PDCs using 10683 gene expressions after removing low expressed genes. (**b**) Expression levels of CCND1 and AXIN1 in 6 matched cell lines, primary HCCs and PDCs. (**c**) Expression trends for 10683 genes between the matched cell lines and primary HCCs. Randomly selected cell lines and primary HCCs were paired to calculate the random background correlations. (**d**) Pearson correlation coefficients between cell lines and PDCs using 10683 gene expressions. Matched, pairs of the matched HCC cell lines and PDCs. Unmatched, pairs of the unmatched HCC cell lines and PDCs. **p < 0.01 by Mann-Whitney test. (**e**) Volcano plot shows DEGs between the matched cell lines and PDCs. Red indicates the significant DEGs.

To determine whether HCC cell lines were similar to matched primary HCCs at the transcription level, we performed RNAseq for all cell lines and PDCs and 6 matched primary HCCs. RNA for primary HCCs CLC5T, CLC6T and CLC7T were degraded and not qualified for RNAseq. By unsupervised hierarchical clustering, we found that the matched cell lines, primary HCCs and PDCs clustered together (Fig. 6a), demonstrating their overall similarities in gene expression patterns. Correlation matrix also showed higher correlation coefficients (Supplementary Fig. S6). Illustrated by examples of 2 genes (CCND1 and AXIN1), expression trends in HCCs (blue curves) were highly correlated with those in matched cell lines (red curves) with Pearson correlation coefficient r = 0.81 for CCND1 and 0.82 for AXIN1 (Fig. 6b). Interestingly, CLC11 harbored *CCND1* amplification and showed higher CCND1 expression levels than CLC13 and CLC16 which had no *CCND1* amplification. Homozygous deletion of *AXIN1* resulted in low gene expression levels in CLC11. More generally, Pearson correlation coefficients showed that expression trends for all the genes were highly correlated between matched cell lines and primary HCCs (Fig. 6c, p < 2.2 × 10^−16^ compared to random pairs). Around 75% of genes showed positive correlations in expression levels between cell lines and matched primary HCCs. These results indicated that HCC cell lines maintained gene expression patterns of primary HCCs.

Notably, hierarchical clustering revealed that cell lines were highly correlated with paired PDCs (Fig. 6a). Pearson correlation coefficients showed that matched HCC cell lines and PDCs were significantly correlated with each other (Fig. 6d). Moreover, there were no differentially expressed genes (DEGs) with a stringent threshold of *P* value < 0.05 where *P* values were corrected for multiple hypothesis testing using Benjamini-Hochberg method. After setting a loose threshold of the uncorrected *P* value to 0.05, only 88 DEGs were detected with a fold change >2 or <1/2 (Fig. 6e, Table S10). No signaling pathways were significantly enriched using these DEGs. The low number of DEGs and large *P* values indicated that the matched HCC cell lines and PDCs had transcriptomes highly similar to each other.

## Discussion

By analyzing HCC cell lines, PDCs and primary cancers at the genome and transcriptome levels, the present work showed that *in vitro* cultured HCC cells largely remained the intrinsic genetic hallmarks of primary HCCs. In line with this finding, previous studies demonstrated that cancer cell lines shared several important mutations with the primary cancers^3^. However, it was not determined whether the genetic alteration landscape was retained in cancer cell lines. Our data clearly showed that HCC cell lines retained the whole genomic landscape of primary HCCs (Figs 3 and 4). During the establishment of HCC cell lines, only a small number of protein-altering mutations were additionally detected in cell lines. No mutations were found to occur repeatedly in cell lines, and importantly, no driver gene mutations in HCCs were additionally accumulated in cell lines. Druggable gene alterations in primary HCCs were also retained in cell lines, further highlighting translational values of these cell lines. Because of technical limitations of copy number detection for WGS, copy number analyses may be prone to false positive^33^. Nonetheless, our data showed overall similarities in CNAs and aneuploidies between matched cell lines and primary HCCs, including *CCND1-FGF19* amplification and *AXIN1* deletion. Moreover, HBV integration sites found in primary HCCs were consistent with matched HCC cell lines. In addition to genetic characterization, we performed transcriptomic comparison between cell lines and primary HCCs. Notably, the specific expression patterns of primary HCCs were preserved in matched cell lines (Fig. 6a). The systematic comparisons provide a solid rationale showing that HCC cell lines could be used as an *in vitro* model for primary HCCs.

It should be noted that additional genetic changes were detected in cell lines and PDCs. By STR analysis (Table S3) and WGS, we found loss of chromosome Y (LOY) in three cell lines (CLC2, 7 and 16). Interestingly, our data were consistent with one previous study in which 18 out of 21 male liver cancer cell lines showed LOY^34^. LOY could happen during the culture, or in the primary HCCs already. For example, previous studies have demonstrated LOY in aged tissues and cancers^35^,^36^. Although it is unclear whether LOY has effect on liver tumorigenesis, it would be interesting to further test whether it imposes a functional effect on cancer cells. Several possibilities may explain cell line-specific mutations, including that (i) a minor cancer cell population of the primary HCC tissues were enriched during *in vitro* culture^5^,^6^, (ii) mutations occurred randomly during cell line establishment, and (iii) HCC cells acquired additional mutations to suit the *in vitro* culture conditions. For the possibility (i), detecting mutations in a minor cell population (<1%) is challenging for WGS due to sequencing depth^37^. We also detected genetic alterations in PDCs that were discordant from matched primary HCCs and cell lines. These alterations might indicate cell population selection during the initial primary culture and long-term culture^5^,^6^. To accurately characterize cell line- and PDC-specific mutations, it requires ultra-deep (sequencing at >1,000X) and multi-region sequencing. For this reason, it was impossible to completely exclude the pre-existence of cell line-specific mutations in primary HCCs. For possibility (ii), 86% of cell line-specific mutations were not enriched in reported signaling pathways related to cancer (Fig. 5c). Moreover, we did not detect cell line-specific mutations occurred in the reported driver genes for HCC. For the possibility (iii), we found that 14 out of 98 mutant genes were enriched in cell cycle and extracellular matrix. This might be because high concentrations of serum and growth factors in culture medium usually stimulate fast proliferation of cancer cells, and *in vitro* cultures lack the proper extracellular matrix presented *in vivo*^32^. An organoid-based culture system that provides the microenvironment rich in extracellular matrix and stromal cells may overcome this challenge^38^,^39^. Be that as it may, the establishment of cell line appear to have limited effects on major cancer hallmarks.

During the establishment of cell lines, we analyzed PDCs at early passages. Due to short-term culture, PDCs are often taken as an *in vitro* model that highly recapitulate the molecular and phenotypic characteristics of primary cancers^11^. However, PDC models are limited to early passages and thus not appropriate for the large-scale drug screening, which requires a large number of cells and comprehensive characterizations. In this study, we specifically compared the genomes and transcriptomes between cell lines and PDCs. HCC cell lines and matched PDCs were similar in mutation landscapes, copy number profiles, HBV integrations and transcriptomes. Driver alteration patterns were highly concordant between HCC cell lines and PDCs. Notably, mutations in genes involved in extracellular matrix and cell cycle observed in cell lines were present in PDCs, suggesting that cancer cells had already adapted these alterations for *in vitro* culture at early passages. It is true that additional few mutations occurred from PDC to cell line, but nearly all of these mutations were unrelated to cancer driver genes. HCC cell lines used in our study were at passages 20–38. Over-passaged cells may introduce much more alterations^4^. It is important to maintain cancer cell lines in standard culture conditions and within proper passages to obtain stable genome and transcriptome. Our results highlight the genetic similarity of the two *in vitro* models and suggest that HCC cell lines may have the potential comparable to that of PDCs in modeling primary HCCs and make drug screening more convenient.

In summary, we showed that HCC cell lines largely represent the mutational and transcriptomic landscapes of matched primary HCCs. These genetically validated HCC cell lines will be deposited in public cell line banks and provide new models for HCCs in functional screening of new therapeutic targets and drug development. With advances in novel technologies, such as HCC organoids, more HCC cell lines would be established at high success rates^38^,^40^. A large panel of HCC cell lines with different mutational profiles would cover a great portion of genomic heterogeneity of primary HCCs. Careful characterization would determine whether these cell lines mimic responses of primary HCCs to different drugs and may lead to a powerful cell line-based platform for precise treatment of HCCs^1^.

## Methods

### Sample collection and cell line establishment

All the fresh cancer tissues were surgically resected from Chinese hepatocellular carcinoma patients with the informed consent. The study was approved by the ethical committee of the participating hospitals and methods were carried out in accordance with the approved guidelines. HCC tissues were rinsed twice with PBS. Both necrotic tissues and apparently normal tissues were discarded. The remainder were finely minced with scissors to small fragments (1 to 2 mm in diameter) and digested by 0.1% Collagenase Type IV (Gibco) in PBS for 30 min at 37 °C with shaking occasionally in a 15 mL or 50 mL centrifuge tube (Corning). Then cell suspensions were filtered by 70 μm cell strainer to remove large fragments and centrifuged consecutively at 1000 rpm, 800 rpm and 600 rpm for 5 min, respectively. Cancer cells were resuspended using primary culture medium and transferred to collagen-coated dishes for culture overnight in a humidified incubator at 37 °C with 5% CO_2_. The primary culture medium were based on RPMI1640 (HyClone), supplemented with 10% fetal bovine serum (FBS, HyClone), 110 μg/mL sodium pyruvate, 10 μg/mL insulin, 5.5 μg/mL transferrin, 40 ng/mL EGF and 6.7 ng/mL sodium selenite (Gibco). On the next day, nearly all the adherent cells showed epithelial-like morphology. Wash with PBS once and change fresh primary culture medium to remove dead cells and blood cells. The primary culture medium were then refreshed every three days. During cell line establishment, epithelial cells were frequently contaminated by fast-growing fibroblast cells. Fibroblast were removed by differential trypsination or scratch by pipetting tips. Because not all the cancer cells from patients overexpress typical cancer or epithelial markers, such as EpCAM, we haven’t sorted isolated cells using these markers to enrich cancer cells. Instead, we observed that during the first several days, nearly all of the adherent cells show epithelial morphology. Epithelial clones were pick out to make sure that epithelial cells were purified and avoid fibroblast contamination. Once confluent, epithelial cells were digested by 0.05% trypsin-EDTA for passage at a ratio of 1:3. Established cell lines were maintained in primary culture medium in a humidified incubator at 37 °C with 5% CO_2_. All the cell lines retained continuous proliferation from passage 10. Cells with more than 20 passages were regarded as cell lines. All the cell lines were authenticated by STR analysis and tested for mycoplasma regularly to avoid contamination. For the passages of PDC, we consulted published work and ongoing project with our own experience. In cell line factory project in Broad Institute (http://www.broadinstitute.org/cellfactory#home), cells through passage 5 were highlighted. In recent reports, passage 1–4 were analyzed as PDCs^10^,^12^. These work also emphasized continuous culture to passage 10 to obtain enough cells for analysis. In patient derived models repository supported by NCI, cells at early passages were also kept as PDCs. Therefore, we kept HCC cells around passage 5 as PDCs in our comparison. We also provided the passage numbers of PDCs in Table S2.

### Growth curves for HCC cell lines and PDCs

Cells at different passages were seeded into 12-well plates at a density of 50,000–200,000 cells for different cells. Cell numbers in different wells were counted at Day 1 (24 hours after seeding cells), 3, 5, 7.

### Histology of primary HCC tissues

HCC tissues were fixed overnight and stained with haematoxylin and eosin for pathological evaluation as previously described^41^.

### Whole genome sequencing and variant calling

Genomic DNA was extracted using Qiagen DNeasy kit. Sequencing library was constructed and paired-end 150 bp read length sequencing was performed on Hiseq X TEN platform according to the manufacturer’s instructions to achieve mean sequencing depth of >30. Sequencing reads were mapped to hg19 human reference genome by Isaac Alignment software with default parameters to obtain BAM files. Isaac Variant Caller was used to call variants with quality scores GQX >30 and generate the filtered VCF files and raw Genome VCF (gVCF) files. SNVs and InDels with low quality were filtered out from VCF files but retained in gVCF files. Considering the relatively low accuracy for InDel identification, we only considered SNVs for the downstream analysis. ANNOVAR^42^ was used for annotation of SNVs. Since no normal tissues were sequenced in this study, we implemented the commonly used pipeline in cell line analysis to call the putative somatic mutations^43^,^44^. SNVs were filtered against the known germline SNV databases (dbSNP135 Non Flagged set, 1000 Genomes Project 2014 October release and ESP6500). SNVs present in these databases were regarded as germline SNVs while the remaining were somatic mutations. For analysis of protein-altering mutations, we curated those which were marked as low quality in gVCF files during SNV calling but passed the filtration in other matched samples.

### Sanger sequencing of protein-altering somatic mutations

Mutation status of TP53, AXIN1, APC, CDKN2A and TERT promoter were confirmed by PCR and Sanger sequencing (Table S5). TP53 mutations and AXIN1 mutation were filtered out in WGS, but validated using Sanger sequencing. For CLC13 HCC, PDC and cell line, TERT promoter mutation in CLC13PDC and APC mutation in CLC13 HCC and cell line were not reported even in raw VCF files, but examined using Sanger sequencing. Primer sequences were provided in Table S11.

### Detection of copy number alterations (CNAs) from WGS

Control-FREEC^20^ was used to normalize read counts from BAM files and obtain the segmentation of smoothed copy number profiles. Segments with copy number above 3 or below 1 were considered as amplifications or deletions. Gene copy numbers were obtained by overlapping CNA segments to the gene locations from UCSC Genome. Copy numbers of chromosome arms were called from the segment files using GISTIC with the “broad length cutoff” parameter 0.8^21^.

### Identification of HBV integration sites from WGS

HBV integration was identified and annotated by Virus-Clip^22^. Sequencing reads were aligned to the HBV genome. Then Virus-Clip extracted soft-clipped reads and mapped the soft-clipped segments to human genome to identify HBV integration breakpoints. Three integration sites which were discordant in WGS among the matched cell lines, primary HCCs and PDCs were validated by Sanger sequencing (Table S6), two of which were not detected by Sanger sequencing and removed for the analysis. Primes sequences were provided in Table S11.

### Hierarchical clustering of genomic data

Whole genome SNVs from 12 samples were merged and unsupervised hierarchical clustering was performed using complete linkage method. Distance between pairs of samples were calculated using Euclidean distance method. Clustering of protein-altering somatic mutations and HBV integration sites was done with the same parameters. For CNAs, gene copy numbers were used as input in the clustering by Manhattan distance method.

### Pathway enrichment analysis of protein-altering mutations

Cell line- or PDC-specific protein-altering mutations were identified by comparing VCF files with curated SNVs of HCC cell lines or PDCs to those of the matched primary HCCs. Mutations which were present in HCC cell lines or PDCs, but absent in primary HCCs, were considered as cell line- or PDC-specific mutations. Pathway enrichment were performed using the online tool of the Molecular Signatures Database (MSigDB) to calculate the overlapping of the gene list to the canonical pathways from the public pathway databases^31^.

### RNA sequencing and data process

Total RNA was extracted using Trizol (Invitrogen) according to the manufacturer’s instructions. Single-end 100 bp read length sequencing was performed on Illumina Hiseq 2000 sequencer. The reads were mapped to the human reference genome (hg19) using Tophat^45^. FPKM (fragments per kilobase of exon per million fragments mapped) values were calculated by Cufflinks using default parameters^45^. Genes with FPKM <1 in more than 12 samples were discarded in downstream analysis. The FPKM values of all the remaining 10683 genes were added with 1 and log2 transformed for heatmap. To remove gene expression changes introduced by culture, gene expression values of *in vitro* cell lines and primary HCCs were separately centered and normalized, and then merged to perform unsupervised hierarchical clustering^39^.

### Identification of differentially expressed genes (DEGs)

Paired Mann-Whitney tests were used to calculate the statistical significance of DEGs from FPKM values. Two thresholds were applied to identify significant DEGs between cell lines and PDCs: 1) fold change >2 or <1/2 and the Benjamini-Hochberg corrected P value < 0.05; 2) fold change >2 or <1/2 and unadjusted P value < 0.05.

### Statistical analysis

Statistical analysis and data visualization were performed using R software and Graphpad Prism software. P values were calculated by statistical methods denoted in each analysis. A two-tailed P < 0.05 was taken to indicate statistical significance unless illustrated otherwise.

## Additional Information

**Accession codes:** Genome data has been deposited at the European Genome-phenome Archive (EGA, http://www.ebi.ac.uk/ega/), which is hosted at the EBI, under accession number EGAS00001001678. RNAseq data have been deposited in NCBI’s Gene Expression Omnibus under the accession number GSE78236.

**How to cite this article**: Qiu, Z. *et al.* Hepatocellular carcinoma cell lines retain the genomic and transcriptomic landscapes of primary human cancers. *Sci. Rep.* **6**, 27411; doi: 10.1038/srep27411 (2016).

## Supplementary Material

Supplementary Information

Supplementary Dataset

## References

[b1] Sharma S. V., Haber D. A. & Settleman J. Cell line-based platforms to evaluate the therapeutic efficacy of candidate anticancer agents. Nature reviews. Cancer 10, 241–253, doi: 10.1038/nrc2820 (2010). 20300105

[b2] Barretina J. *et al.* The Cancer Cell Line Encyclopedia enables predictive modelling of anticancer drug sensitivity. Nature 483, 603–607, doi: 10.1038/nature11003 (2012). 22460905 PMC3320027

[b3] Masters J. R. Human cancer cell lines: fact and fantasy. Nature reviews. Molecular cell biology 1, 233–236, doi: 10.1038/35043102 (2000). 11252900

[b4] Hughes P., Marshall D., Reid Y., Parkes H. & Gelber C. The costs of using unauthenticated, over-passaged cell lines: how much more data do we need? BioTechniques 43, 575, 577–578, 581–572 passim (2007). 10.2144/000112598 18072586

[b5] Drexler H. G. *et al.* p53 alterations in human leukemia-lymphoma cell lines: in vitroartifact or prerequisite for cell immortalization? Leukemia 14, 198–206 (2000). 10637496 10.1038/sj.leu.2401604

[b6] Hartmann C., Kluwe L., Lucke M. & Westphal M. The rate of homozygous CDKN2A/p16 deletions in glioma cell lines and in primary tumors. International journal of oncology 15, 975–982 (1999). 10536182 10.3892/ijo.15.5.975

[b7] Wistuba I. I. *et al.* Comparison of features of human lung cancer cell lines and their corresponding tumors. Clinical cancer research: an official journal of the American Association for Cancer Research 5, 991–1000 (1999). 10353731

[b8] Larramendy M. L. *et al.* Comparative genomic hybridization reveals complex genetic changes in primary breast cancer tumors and their cell lines. Cancer genetics and cytogenetics 119, 132–138 (2000). 10867149 10.1016/s0165-4608(99)00226-5

[b9] De Witt Hamer P. C. *et al.* The genomic profile of human malignant glioma is altered early in primary cell culture and preserved in spheroids. Oncogene 27, 2091–2096, doi: 10.1038/sj.onc.1210850 (2008). 17934519

[b10] Dairkee S. H. *et al.* Partial enzymatic degradation of stroma allows enrichment and expansion of primary breast tumor cells. Cancer research 57, 1590–1596 (1997). 9108465

[b11] Mitra A., Mishra L. & Li S. Technologies for deriving primary tumor cells for use in personalized cancer therapy. Trends in biotechnology 31, 347–354, doi: 10.1016/j.tibtech.2013.03.006 (2013). 23597659 PMC3665643

[b12] Lee J. Y. *et al.* Patient-derived cell models as preclinical tools for genome-directed targeted therapy. Oncotarget 6, 25619–25630, doi: 10.18632/oncotarget.4627 (2015). 26296973 PMC4694854

[b13] El-Serag H. B. & Rudolph K. L. Hepatocellular carcinoma: epidemiology and molecular carcinogenesis. Gastroenterology 132, 2557–2576, doi: 10.1053/j.gastro.2007.04.061 (2007). 17570226

[b14] Llovet J. M. *et al.* Sorafenib in advanced hepatocellular carcinoma. The New England journal of medicine 359, 378–390, doi: 10.1056/NEJMoa0708857 (2008). 18650514

[b15] Zucman-Rossi J., Villanueva A., Nault J. C. & Llovet J. M. Genetic Landscape and Biomarkers of Hepatocellular Carcinoma. Gastroenterology 149, 1226-1239 e1224, doi: 10.1053/j.gastro.2015.05.061 (2015). 26099527

[b16] Schulze K. *et al.* Exome sequencing of hepatocellular carcinomas identifies new mutational signatures and potential therapeutic targets. Nature genetics 47, 505–511, doi: 10.1038/ng.3252 (2015). 25822088 PMC4587544

[b17] Park J. G. *et al.* Characterization of cell lines established from human hepatocellular carcinoma. International journal of cancer. Journal international du cancer 62, 276–282 (1995). 7543080 10.1002/ijc.2910620308

[b18] Ajay S. S., Parker S. C., Abaan H. O., Fajardo K. V. & Margulies E. H. Accurate and comprehensive sequencing of personal genomes. Genome research 21, 1498–1505, doi: 10.1101/gr.123638.111 (2011). 21771779 PMC3166834

[b19] Jia D. *et al.* Genome-wide copy number analyses identified novel cancer genes in hepatocellular carcinoma. Hepatology 54, 1227–1236, doi: 10.1002/hep.24495 (2011). 21688285

[b20] Boeva V. *et al.* Control-FREEC: a tool for assessing copy number and allelic content using next-generation sequencing data. Bioinformatics 28, 423–425, doi: 10.1093/bioinformatics/btr670 (2012). 22155870 PMC3268243

[b21] Mermel C. H. *et al.* GISTIC2.0 facilitates sensitive and confident localization of the targets of focal somatic copy-number alteration in human cancers. Genome biology 12, R41, doi: 10.1186/gb-2011-12-4-r41 (2011). 21527027 PMC3218867

[b22] Ho D. W., Sze K. M. & Ng I. O. Virus-Clip: a fast and memory-efficient viral integration site detection tool at single-base resolution with annotation capability. Oncotarget 6, 20959–20963, doi: 10.18632/oncotarget.4187 (2015). 26087185 PMC4673242

[b23] Totoki Y. *et al.* Trans-ancestry mutational landscape of hepatocellular carcinoma genomes. Nature genetics 46, 1267–1273, doi: 10.1038/ng.3126 (2014). 25362482

[b24] Sung W. K. *et al.* Genome-wide survey of recurrent HBV integration in hepatocellular carcinoma. Nature genetics 44, 765–769, doi: 10.1038/ng.2295 (2012). 22634754

[b25] Forbes S. A. *et al.* COSMIC: exploring the world’s knowledge of somatic mutations in human cancer. Nucleic acids research 43, D805–811, doi: 10.1093/nar/gku1075 (2015). 25355519 PMC4383913

[b26] Rubio-Perez C. *et al.* In silico prescription of anticancer drugs to cohorts of 28 tumor types reveals targeting opportunities. Cancer cell 27, 382–396, doi: 10.1016/j.ccell.2015.02.007 (2015). 25759023

[b27] Finn R. S. *et al.* The cyclin-dependent kinase 4/6 inhibitor palbociclib in combination with letrozole versus letrozole alone as first-line treatment of oestrogen receptor-positive, HER2-negative, advanced breast cancer (PALOMA-1/TRIO-18): a randomised phase 2 study. The Lancet. Oncology 16, 25–35, doi: 10.1016/S1470-2045(14)71159-3 (2015). 25524798

[b28] Sawey E. T. *et al.* Identification of a therapeutic strategy targeting amplified FGF19 in liver cancer by Oncogenomic screening. Cancer cell 19, 347–358, doi: 10.1016/j.ccr.2011.01.040 (2011). 21397858 PMC3061399

[b29] Futreal P. A. *et al.* A census of human cancer genes. Nature reviews. Cancer 4, 177–183, doi: 10.1038/nrc1299 (2004). 14993899 PMC2665285

[b30] Kandoth C. *et al.* Mutational landscape and significance across 12 major cancer types. Nature 502, 333–339, doi: 10.1038/nature12634 (2013). 24132290 PMC3927368

[b31] Subramanian A. *et al.* Gene set enrichment analysis: a knowledge-based approach for interpreting genome-wide expression profiles. Proceedings of the National Academy of Sciences of the United States of America 102, 15545–15550, doi: 10.1073/pnas.0506580102 (2005). 16199517 PMC1239896

[b32] van Staveren W. C. *et al.* Human cancer cell lines: Experimental models for cancer cells *in situ*? For cancer stem cells? Biochimica et biophysica acta 1795, 92–103, doi: 10.1016/j.bbcan.2008.12.004 (2009). 19167460

[b33] Tan R. *et al.* An evaluation of copy number variation detection tools from whole-exome sequencing data. Human mutation 35, 899–907, doi: 10.1002/humu.22537 (2014). 24599517

[b34] Park S. J., Jeong S. Y. & Kim H. J. Y chromosome loss and other genomic alterations in hepatocellular carcinoma cell lines analyzed by CGH and CGH array. Cancer genetics and cytogenetics 166, 56–64, doi: 10.1016/j.cancergencyto.2005.08.022 (2006). 16616112

[b35] Guttenbach M., Koschorz B., Bernthaler U., Grimm T. & Schmid M. Sex chromosome loss and aging: *in situ* hybridization studies on human interphase nuclei. American journal of human genetics 57, 1143–1150 (1995). 7485166 PMC1801353

[b36] Bianchi N. O. Y chromosome structural and functional changes in human malignant diseases. Mutation research 682, 21–27, doi: 10.1016/j.mrrev.2009.02.001 (2009). 19699459

[b37] Sims D., Sudbery I., Ilott N. E., Heger A. & Ponting C. P. Sequencing depth and coverage: key considerations in genomic analyses. Nature reviews. Genetics 15, 121–132, doi: 10.1038/nrg3642 (2014). 24434847

[b38] Gao D. *et al.* Organoid cultures derived from patients with advanced prostate cancer. Cell 159, 176–187, doi: 10.1016/j.cell.2014.08.016 (2014). 25201530 PMC4237931

[b39] van de Wetering M. *et al.* Prospective derivation of a living organoid biobank of colorectal cancer patients. Cell 161, 933–945, doi: 10.1016/j.cell.2015.03.053 (2015). 25957691 PMC6428276

[b40] Liu X. *et al.* ROCK inhibitor and feeder cells induce the conditional reprogramming of epithelial cells. The American journal of pathology 180, 599–607, doi: 10.1016/j.ajpath.2011.10.036 (2012). 22189618 PMC3349876

[b41] Min L. *et al.* Liver cancer initiation is controlled by AP-1 through SIRT6-dependent inhibition of survivin. Nature cell biology 14, 1203–1211, doi: 10.1038/ncb2590 (2012). 23041974

[b42] Wang K., Li M. & Hakonarson H. ANNOVAR: functional annotation of genetic variants from high-throughput sequencing data. Nucleic acids research 38, e164, doi: 10.1093/nar/gkq603 (2010). 20601685 PMC2938201

[b43] Liu J. *et al.* Genome and transcriptome sequencing of lung cancers reveal diverse mutational and splicing events. Genome research 22, 2315–2327, doi: 10.1101/gr.140988.112 (2012). 23033341 PMC3514662

[b44] Mouradov D. *et al.* Colorectal cancer cell lines are representative models of the main molecular subtypes of primary cancer. Cancer research 74, 3238–3247, doi: 10.1158/0008-5472.CAN-14-0013 (2014). 24755471

[b45] Trapnell C. *et al.* Differential gene and transcript expression analysis of RNA-seq experiments with TopHat and Cufflinks. Nature protocols 7, 562–578, doi: 10.1038/nprot.2012.016 (2012). 22383036 PMC3334321

