# Supplementary material for: Hepatocellular carcinoma cell lines retain the genomic and transcriptomic landscapes of primary human cancers
Source: Sci Rep. 2016 Jun 7;6:27411. doi: 10.1038/srep27411 (PMC4895220; doi:10.1038/srep27411)

Hepatocellular carcinoma cell lines retain the genomic and transcriptomic landscapes of primary human cancers

Zhixin Qiu, Keke Zou, Liping Zhuang, Jianjie Qin, Hong Li, Chao Li, Zhengtao Zhang, Xiaotao Chen, Jin Cen, Zhiqiang Meng, Haibin Zhang, Yixue Li, and Lijian Hui

Supplementary information

Supplementary Figure Legends

Supplementary Figure S1. Characteristics of HCC cell lines.

(a) Representative morphologies of 5 HCC cell lines. Scale bars: 100 µm. (b) Expression levels of Albumin and AFP in 9 HCC cell lines, as demonstrated by FPKM values from RNAseq. (c) HBV integration in the genomes in 9 HCC cell lines by PCR amplification of segments of HBV genome. Among 9 cell lines, 7 cell lines showed HBV integration in this experiment. CLC2 were derived from HCV-positive and HBV-negative patients, and showed no HBV integration. We didn't detect HBV integration for CLC16 in PCR, but in unbiased WGS analysis. The segment of HBV genome in PCR amplification might be absent in CLC16.

Supplementary Figure S2. Validation of TP53 mutations by Sanger sequencing.

(a) Variant allele frequencies (VAFs) of TP53 mutations in raw gVCF files from whole genome sequencing analysis in the cell lines, primary HCCs and PDCs. Chr, chromosome. Ref/Alt reads, counts of reads supporting reference allele or alternative allele, respectively. (b) Sanger sequencing

of three TP53 mutations identified in three HCC cell lines by whole genome sequencing. Red indicates the mutation site. Hg19, human reference genome build 19.

Supplementary Figure S3. Protein-altering somatic mutations in the matched cell lines, primary HCCs and PDCs.

(a) The number of protein-altering somatic mutations in the matched cell lines, primary HCCs and PDCs. (b-e) Venn diagrams show the overlapping of protein-altering somatic mutations in matched cell lines, primary HCCs and PDCs.

Supplementary Figure S4. Comparison of chromosome arm copy number alterations.

Paired comparison of the matched cell lines, primary HCCs and PDCs using copy numbers at the chromosome arm level to reflect the overall similarities in aneuploidy.

Supplementary Figure S5. Cell line-specific and PDC-specific protein-altering mutations compared to the matched primary HCCs.

(a-d) Venn diagrams show the overlapping of cell line-specific mutations (red) and PDC-specific mutations (blue) compared to the matched primary HCCs. Highlighted genes represent mutant genes associated with extracellular matrix and cell cycle regulation in Fig. 5d.

Supplementary Figure S6. Correlation matrix of the matched cell lines, primary HCCs and PDCs.

Heatmap and dendrogram shows Pearson correlation coefficients matrix calculated using 10683 gene expressions in Fig. 6A. Red, positive correlation; blue, negative correlation.

Supplementary Figure S1

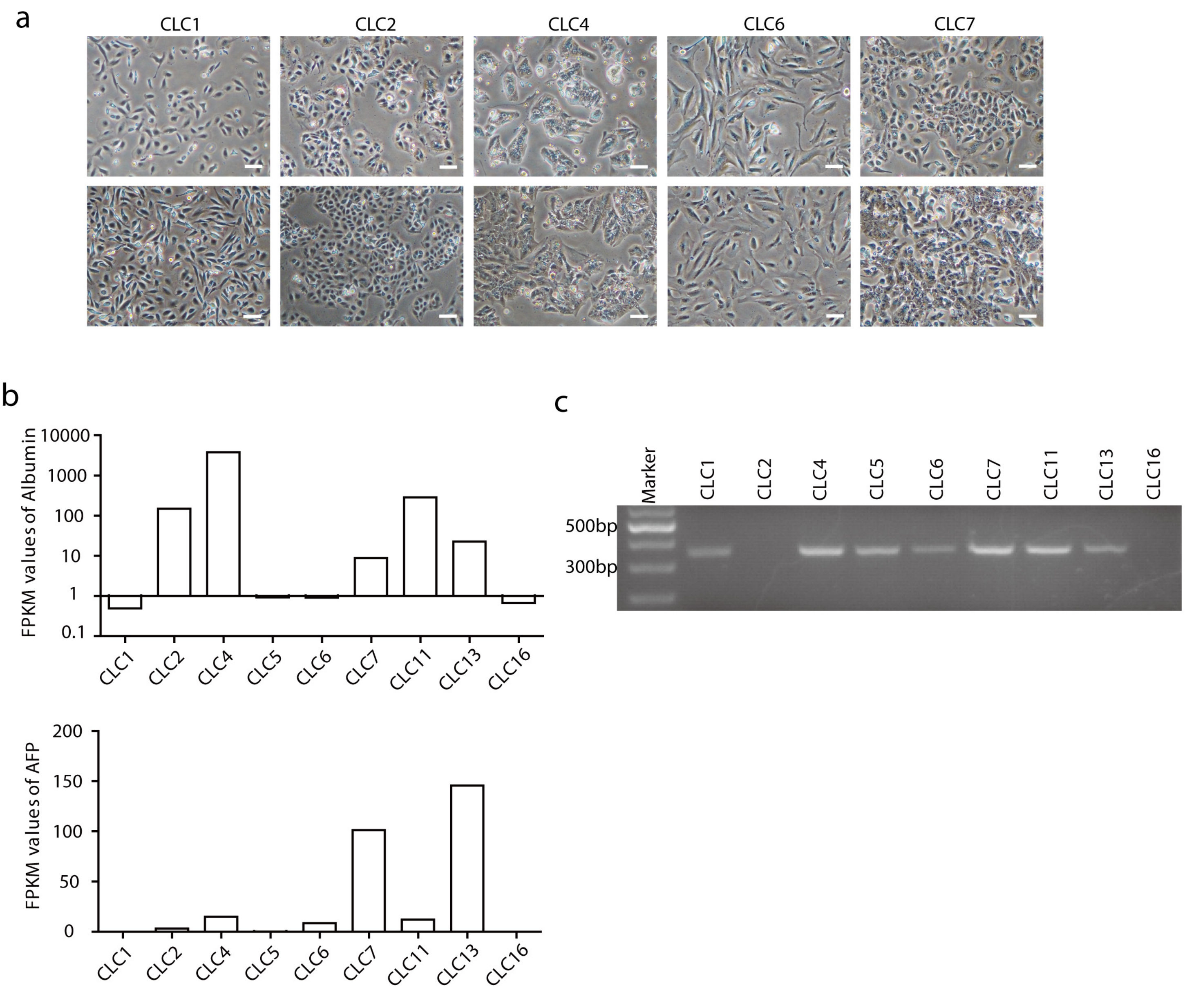

Supplementary Figure S2

a

Sample	Mutation	WGS	Ref reads	Alt reads	VAF
CLC5T	chr17:7579485:C-A	No mutation	1	12	0.923077
CLC5PDC		No mutation	1	15	0.9375
CLC5		Mutation	0	18	1
CLC11T	chr17:7577568:C-A	No mutation	1	15	0.9375
CLC11PDC		Mutation	0	18	1
CLC11		Mutation	0	14	1
CLC13T	chr17:7579373:C-T	No mutation	1	17	0.944444
CLC13PDC		No mutation	1	9	0.9
CLC13		Mutation	0	20	1

b

chr17:7579485:C-A

Hg19 Genome GGAGCAGCCTCTGGCATTCTG
CLC5T GGAGCAGCCTATGGCATTCTG
CLC5PDC GGAGCAGCCTATGGCATTCTG
CLC5 GGAGCAGCCTATGGCATTCTG

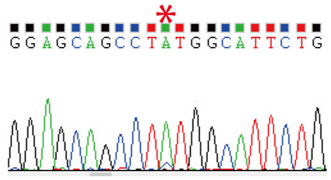

chr17:7577568:C-A

Hg19 Genome GGAACTGTTACACATGTAGTT
CLC11T GGAACTGTTAAACATGTAGTT
CLC11PDC GGAACTGTTAAACATGTAGTT
CLC11 GGAACTGTTAAACATGTAGTT

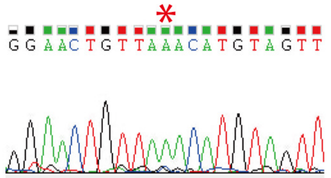

chr17:7579373:C-T

Hg19 Genome ACCGTAGCTGCCCTGGTAGGT
CLC13T ACCGTAGCTGTCCTGGTAGGT
CLC13PDC ACCGTAGCTGTCCTGGTAGGT
CLC13 ACCGTAGCTGTCCTGGTAGGT

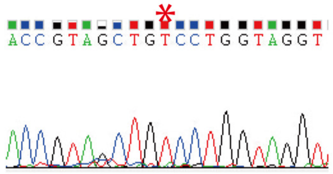

Supplementary Figure S3

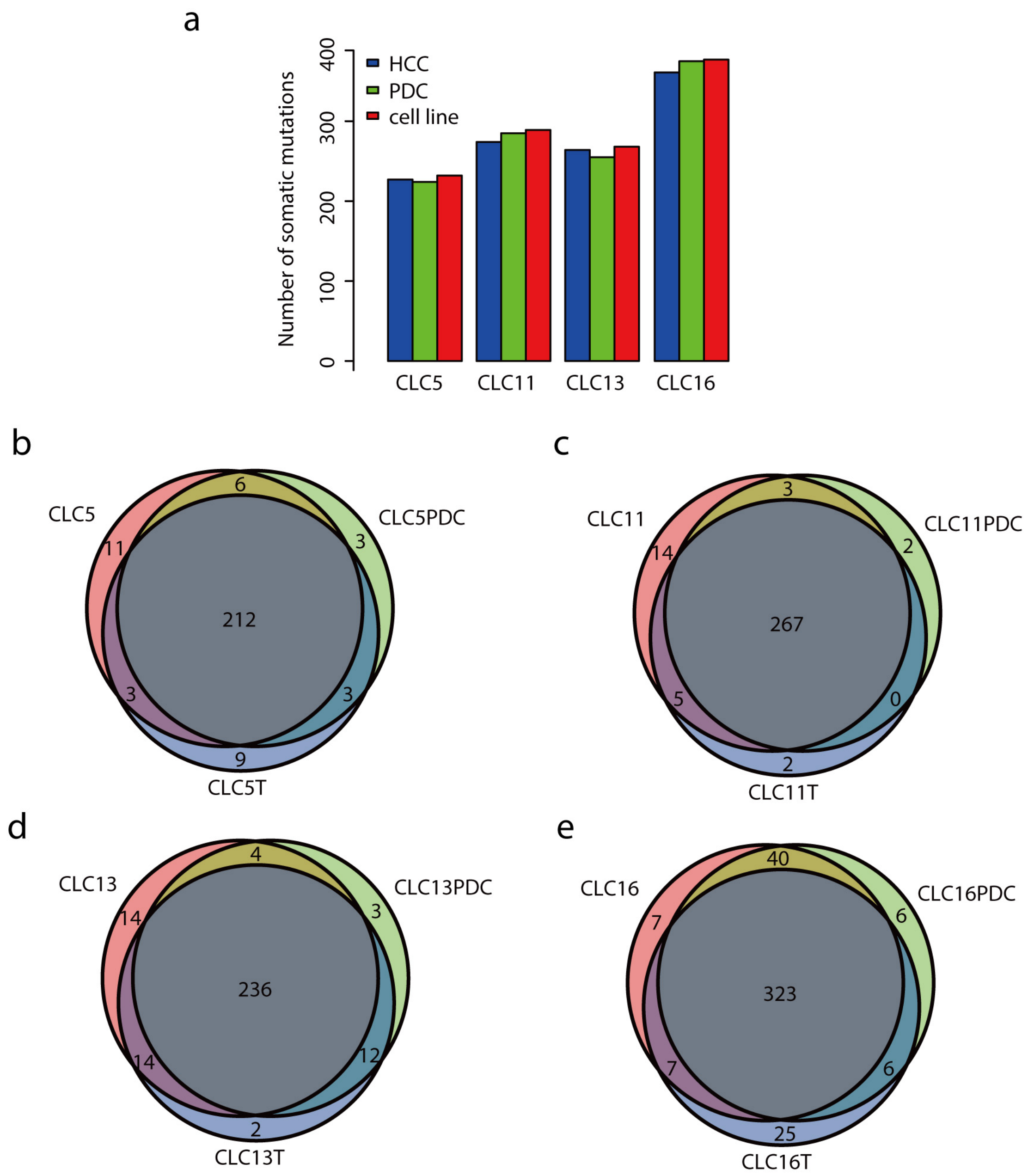

Supplementary Figure S4

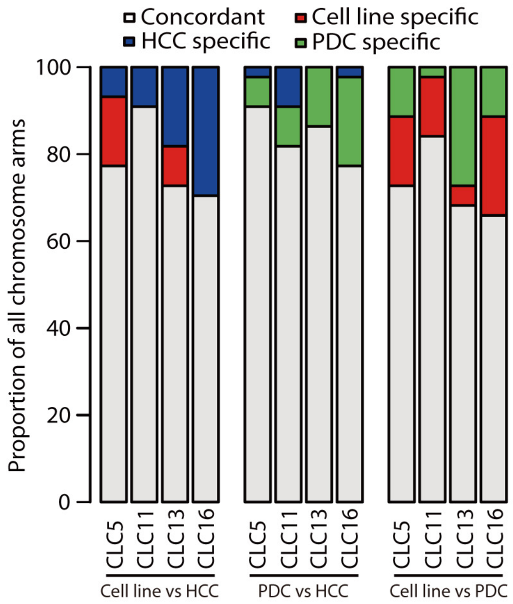

Supplementary Figure S5

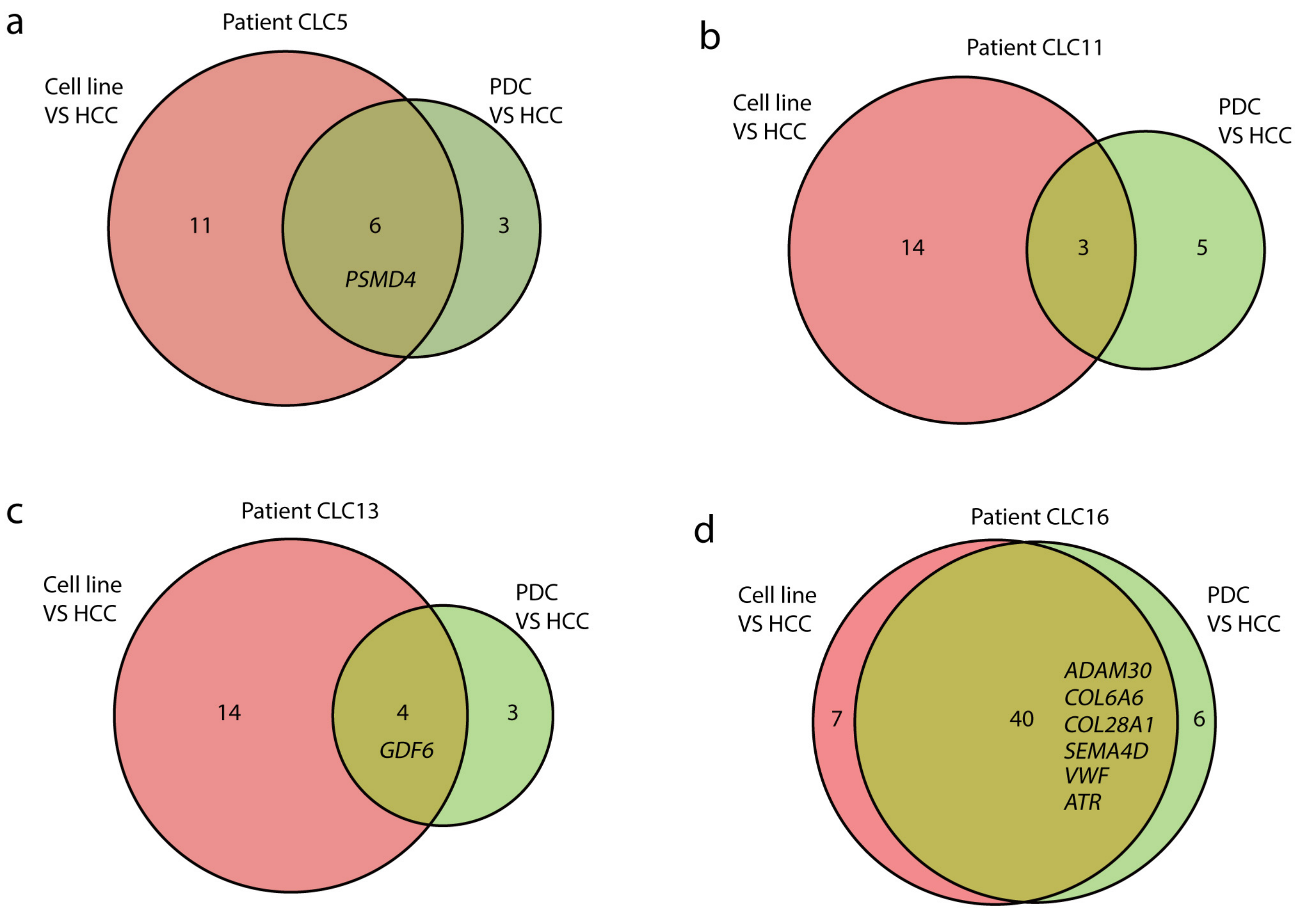

Supplementary Figure S6

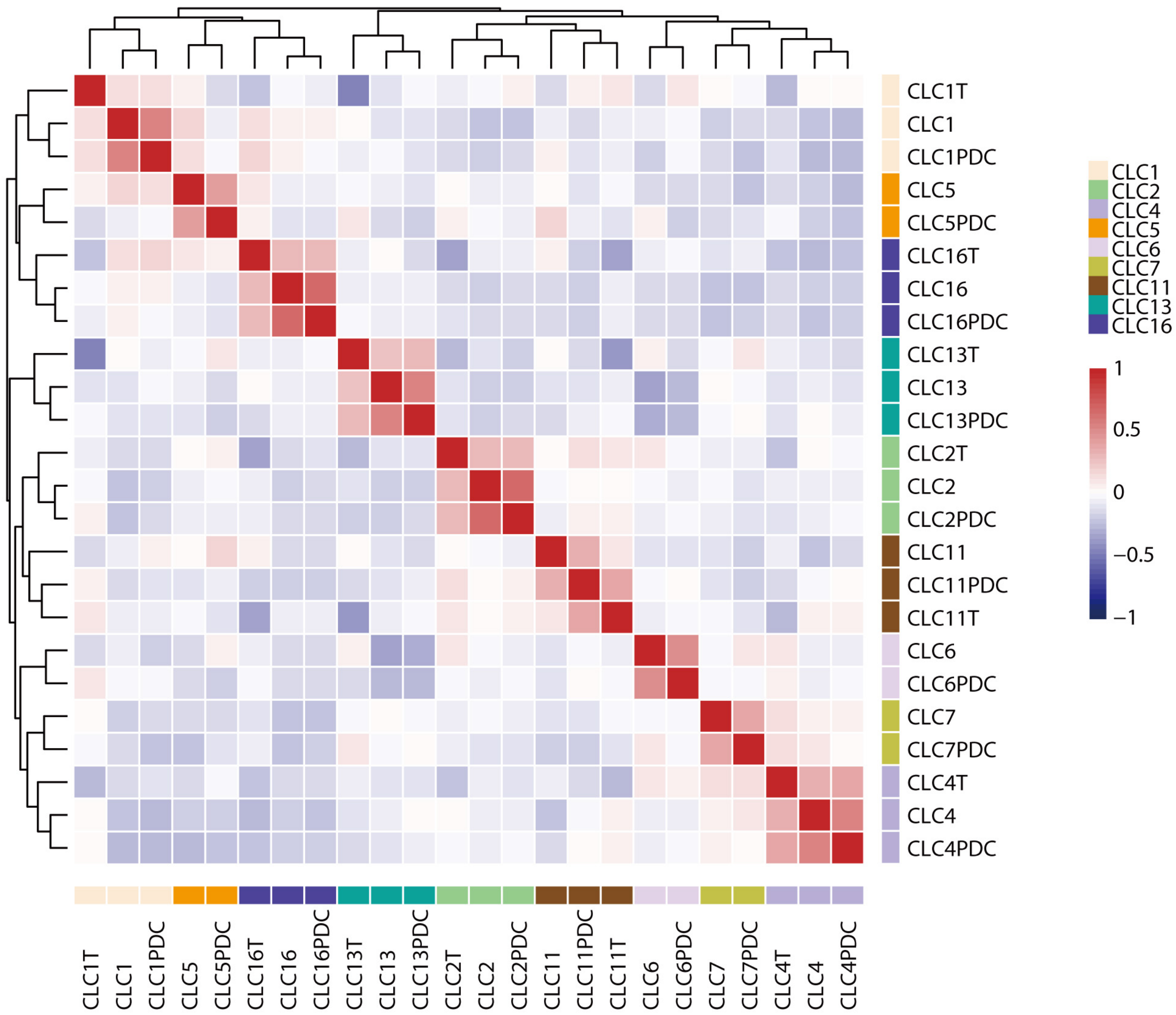

Supplement: Supplementary Information [file srep27411-s1.pdf]
